# The translational value of calcium pyrophosphate deposition disease experimental mouse models

**DOI:** 10.3389/fmed.2024.1417318

**Published:** 2024-05-23

**Authors:** Roberto Luisetto, Anna Scanu

**Affiliations:** ^1^Experimental Surgery Research Center, Department of Surgery, Oncology and Gastroenterology-DISCOG, University of Padova, Padova, Italy; ^2^Department of Women's and Children's Health-SDB, University of Padova, Padova, Italy; ^3^Departement of Neuroscience-DNS, University of Padova, Padova, Italy

**Keywords:** calcium pyrophosphate, pyrophosphate, arthritis, mouse models, translational models, inflammation, CPPD

## Abstract

The deposition of calcium pyrophosphate (CPP) crystals in joint tissues causes acute and chronic arthritis that commonly affect the adult and elderly population. Experimental calcium pyrophosphate deposition disease (CPPD) models are divided into genetically modified models and crystal-induced inflammation models. The former do not reproduce phenotypes overlapping with the human disease, while in the latter, the direct injection of crystals into the ankles, dorsal air pouch or peritoneum constitutes a useful and reliable methodology that resembles the CPP induced-inflammatory condition in humans. The translational importance of the induced model is also strengthened by the fact that the key molecular and cellular mediators involved in inflammation are shared between humans and laboratory rodents. Although, *in vivo* models are indispensable tools for studying the pathogenesis of the CPPD and testing new therapies, their development is still at an early stage and major efforts are needed to address this issue. Here, we analyze the strenghts and limitations of each currently available CPPD *in vivo* model, and critically discuss their translational value.

## Introduction

Crystal deposition in the articular and periarticular tissues causes arthropathies that commonly affect the adult and elderly population. Among the most common and well-known of these crystals is calcium pyrophosphate (CPP), which is the causative agent of inflammation and joint damage. The clinical manifestations of calcium pyrophosphate deposition disease (CPPD) can range from asymptomatic tissue calcification to acute or chronic arthritis, and in some cases it may be associated with other conditions, such as osteoarthritis (1).

Although CPPD is a prevalent rheumatic musculoskeletal disease, the precise processes that lead to crystal formation and the molecular mechanisms involved in inflammation remain unclear. The use of experimental murine models has proven to be an indispensable tool to study the pathogenesis of this disease and efficacy of potential treatments; however, their translational value with respect to the corresponding human pathology has to be carefully evaluated considering the strengths, species-specific differences, and possible critical points during the experimental design. Since rodents do not develop crystal deposition diseases spontaneously, experimental modeling is performed either through transgenic strains that reproduce characteristics observed in patients or by exploiting the inflammatory potential of crystals directly injected into joint tissues, peritoneum, artificial dorsal pouches, or bone marrow of wild-type animals (2–5).

### Genetically modified models

So far, it has not yet been possible to create laboratory rodents that spontaneously develop CPP crystal-induced arthritis or in which crystal formation is observed.

Since the presence of extracellular inorganic pyrophosphate (ePPi) in cartilage tissues is critical for CPP crystal formation (6, 7), knockout (KO) mice for enzymes or other proteins involved in PPi metabolism and calcification processes were generated. However, despite KO mice for TNAP (Tissue-nonspecific Alkaline Phosphatase, (Akp2^−/−^)), Phospho1 (phosphoethanolamine/phosphocholine phosphatase 1, (Phospho1^−/−^)) and osteopontin (OPN) are characterized by high circulating levels of ePPi, they did not show signs of arthritis or a phenotype compatible with CPPD (8–10). The absence of articular involvement was also confirmed in double knock out Akp2^−/-^OPN^−/−^ and Akp2^−/-^Phospho1^−/−^ mice, generated to alter the concerted action of ePPi regulators (10, 11). Furthermore, these knock-out models characterized by high levels of ePPi exhibit high embryonic lethality and shortened lifespan, severe disturbances in bone mineralization, and skeletal abnormalities that did not overlap with the human condition (8, 12).

Several hypotheses may be formulated to explain why CPP crystals are not deposited in mice, which should be considered when using genetically conditioned models. For instance, yet unknown molecular mechanisms underlying the regulation of ePPi level, or the thickness of the articular cartilage layers on average 50-fold thinner than in humans, together with different temperatures and tissutal pH could prevent the crystal formation. Furthermore, the relatively short lifespan of the laboratory mouse may not allow enough time for crystal formation to occur.

Unlike the models described above in which mutations are induced artificially, the progressive ankylosis (ank/ank) mouse is a strain with a spontaneous mutation in the Ank gene, that shows progressive impairment of joint mobility and extremely early arthritis with progressive unrelenting ectopic calcification and vertebral fusion conducing to death within 6 months of age (13). The progressive ankylosis gene (ank) encodes a transmembrane protein that regulates the cellular efflux of ATP. Interestingly, several missense mutations have been described in familial CPPD disease and up-regulation of ANK protein expression was found in articular tissues from patients with CPP deposition (14, 15). In particular, this human condition is associated with increases in ePPi and presumed gain-of-function of ANK (14). However, the ank/ank murine model displays important differences compared to human patients with CPPD. Indeed, mice develop a disorder clinically, radiographically, and histologically more similar to human spondyloarthropathies than to CPPD (16), and histopathological evaluation demonstrated significant deposition of hydroxyapatite (HA) crystals but not CPP in the affected joints. Furthermore, this model is characterized by a loss-of-function mutation in ANK, an increase in intracellular PPi (iPPi) that triggers HA crystal deposition, and a decrease in ePPi (2, 17).

Another naturally occurring mutant mouse model linked to altered mineralization is the “tiptoe walking” (ttw/ttw) mouse. This model shows a defective expression of NPP1, the main enzyme that generates PPi, caused by a nonsense mutation in the corresponding gene (18). The ttw/ttw mice exhibit marked postnatal ectopic ossification, including the development of progressive intervertebral ankylosis, arterial calcification, as well as spontaneous increased bone formation process and calcification of articular cartilage in joints (18, 19). However, although calcium crystal deposition was observed in the joint tissues of these mutant mice, the presence of CPP was not evidenced (19, 20). This may be due to reduced levels of ePPi, which have been determined in ttw/ttw and other NPP1-deficient mice (21).

The most widely used genetically modified mouse models to reproduce PPi metabolism and calcification processes are summarized in Table 1.

**Table 1:** Genetically modified mouse models to reproduce PPi metabolism and calcification processes.

Mouse Model	Phenotype	PPi levels	Outcomes	Strenghts	Weakness	References
Akp2^−/−^	Early axial skeletal mineralization defects and impaired condrocytes differentiation	ePPi↑	Skeletal development, PPi metabolism	Model of hypophosphatasia and osteopenia	No CPP deposition in joints, no arthritis	(8)
Phospho1^−/−^	Cartilage growth abnormalities, spontaneous fractures, osteomalacia, scoliosis	ePPi↑	Endochondral ossification, skeletal development	Model of hypophosphatasia	No CPP deposition in joints, no arthritis	(10)
OPN^−/−^	Normal skeleton	ePPi↑	Bone mineralization, PPi metabolism	Crosstalk between OPN and phosphatases	Normal phenotype	(9)
Akp2^−/-^OPN^−/−^	Mild defects in bone mineralization	ePPi↑	Bone mineralization and PPi metabolism	Crosstalk between OPN and phosphatases	No CPP deposition in joints, limited lifespan, no arthritis	(9)
Akp2^−/-^Phospho1^−/−^	Impaired skeletal mineralization	ePPi↔	Skeletal calcification and PPi metabolism	Crosstalk PPi enzymes	No CPP deposition in joints, limited lifespan, no arthritis	(10)
ank/ank	Early arthritis and progressive ankylosis	iPPi↑ ePPi↓	Calcification and PPi metabolism	Involvement of ANK transporter	No CPP deposition in joints, model based on ANK loss-of-function	(2)
ttw/ttw	Severe disorders in bone mineralization	ePPi↓	Bone mineralization and PPi metabolism	Model of spondyloarthropathy and ossification of posterior longitudinal ligaments of spine	No CPP deposition in joints, no arthritis	(18, 19)

### Crystal-induced inflammation models

In the absence of spontaneous models, CPP crystal injection can be used to reproduce inflammatory responses similar to those of patients with CPPD. For example, injection of CPP crystals in the ankle causes joint swelling that develops after a few hours, progressively increases, and then declines within a few days (22). Similar conditions are also observed after injection of monosodium urate (MSU) crystals, which are responsible for gout. However, unlike MSU crystal-induced inflammation model in which ankle swelling is maximal at 24 h and completely resolves within 5 days (23), swelling induced by CPP injection peaks after 48 h and persists longer, remaining higher than control even after 6 days (22). Interestingly, this reflects the clinical course in patients. Indeed, untreated gout flares commonly remit spontaneously within a few days, while acute CPP crystal arthritis may persist longer and usually resolve within 1 to 3 weeks (24).

The strength points of this model include: (1) similarity between the human and mouse anatomical joint structure, (2) the acute attack following CPP stimulation induces, in both species, non-specific lesions such as edema, inflammatory infiltrate characterized mainly by neutrophils and macrophages, and (3) in the most severe exacerbations, it is also possible to observe muscle, cartilaginous injury, and synovial hyperplasia (25). Furthermore, swelling of the paw, ankle or knee tissues is extremely rapid and is usually measured using a precision caliber and constitutes a useful parameter for quantifying edema and inflammation. The main limitation of the model is related to the technical difficulty of carrying out the procedure. Crystals are injected into the ankle at the tibio-tarsal joint, but it can be difficult to establish whether it occurred correctly within the joint or in the peri-articular tissues. Of note, despite the importance of the synovial fluid (SF) collection and analysis in clinical diagnosis in human patients (1), these are generally excluded from experimental research designs due to the low amount of SF, which is estimated to be less than 1 μL in mouse knee and ankle joints (26, 27).

The air pouch, crystal-induced peritonitis and pleuritis are experimental models that do not directly involve the joint tissues. In these contexts, the injection of crystals into the cavity triggers a rapid infiltration of neutrophils and macrophages and an increase in the volume of exudate which is also enriched with inflammatory mediators that can be quantified (28–32).

The dorsal air pouch model, described for the first time by Selye, is an easily performed procedure and widely used for the study of several anti-inflammatory and antirheumatic agents because it is able to mimic the synovial environment (30, 33).

However, repeated injection of sterile air into the back of mice may cause microtraumas that trigger an inflammatory response.

### CPP crystal-induced inflammation as an experimental model for sterile inflammation

Mice and humans share about 80% of the genetic background and, by comparing genome-wide transcriptional data, Shay et al. demonstrated that both species have essentially the same molecular pathways involved in inflammation and in innate and cellular immune system (34). These findings make experimental models of crystal-induced inflammation a powerful tool for studying sterile inflammation. It is known that crystals, through the activation of inflammasome NLRP3 and TLRs and production of IL-1β, induce massive expression of pro-inflammatory cytokines such as IL-8, CCL2, IL-6, TNF-α as well as other inflammatory mediators (30, 35–39). In this context, several knock-out/knock-in and transgenic mice have been produced for several factors involved in the NRLP3 inflammatory pathway. Generally, knock-out animals are resistant to inflammation induced by crystal injection, making them a useful system for pathophysiological studies rather than for testing new therapies. For example, mice deficient in various key proteins in the inflammasome complex NRLP3 or the IL-1 receptor (IL-1R) are fertile and viable, but did not show inflammatory changes when isolated macrophages were stimulated, or animals were intraperitoneally injected with CPP crystals (38). Interestingly, opposite conditions were observed after injection of BCP crystals, which are frequently found in OA joints. Indeed, i.a. administration of BCP crystals in the knee of NLRP3 and ASC, IL-1β or IL-1α deficient mice showed similar inflammation compared to WT mice (40), thus suggesting that different crystals can trigger distinct inflammatory pathways and careful consideration should be taken when choosing the most appropriate animal model.

TLR-4 is another key factor shared between the two species. Indeed, this is mainly expressed in myeloid cells in both humans and mice and has high sequence homology in the promoter sequence and receptor transcript (41). However, unlike humans, murine TLR-4 is also significantly expressed in skeletal muscles, where CPP can accumulate during experimental procedures; this may explain the reason of persistent edema observed after injection of crystals into the ankle of mice (22, 42).

The use of murine models of crystal-induced inflammation is further corroborated by the fact that mice and humans do not differ in total leukocyte count in peripheral blood. Furthermore, although in healthy mice lymphocytes are the predominant leukocyte making up 70 to 80%, while neutrophils generally comprise 20 to 30% of the white blood cell differential count, in acute inflammatory conditions the lymphocyte/neutrophil ratio is rapidly reversed, thus reproducing the typical pattern of acute inflammation observed in humans (43–45).

These events occur despite the fact that the homolog of human IL-8, the main chemokine regulating neutrophil recruitment, has not been detected in rodents (46). The absence of IL-8 in mice seems compensated for by the ligands CXCL1/KC, which bind the murine CXCR2 receptor and attract immune cells to inflamed tissues (30, 47).

## Conclusion

In conclusion, animal models are needed to understand the pathophysiology of CPPD, but their development is still at an early stage. In this context, some general critical issues have to be considered in the creation of animal models that may interfere with their translational value. For example, *in vivo* experiments to study CPPD involve the use of healthy and pathogen-free animals of inbred strains with many homozygous mutations, whereas the human population is highly heterogeneous in terms of genetic background and individual clinical history. Furthermore, there are often significant age- and sex-related biases in exclusively using young and male mice that scarcely overlap with the epidemiology of CPPD. It usually affects elderly subjects and is slightly more common in women than in men.

Currently available genetically conditioned models contribute to understanding of some aspects of disease but are far to mimic the human CPPD pathology.

**Figure 1:**
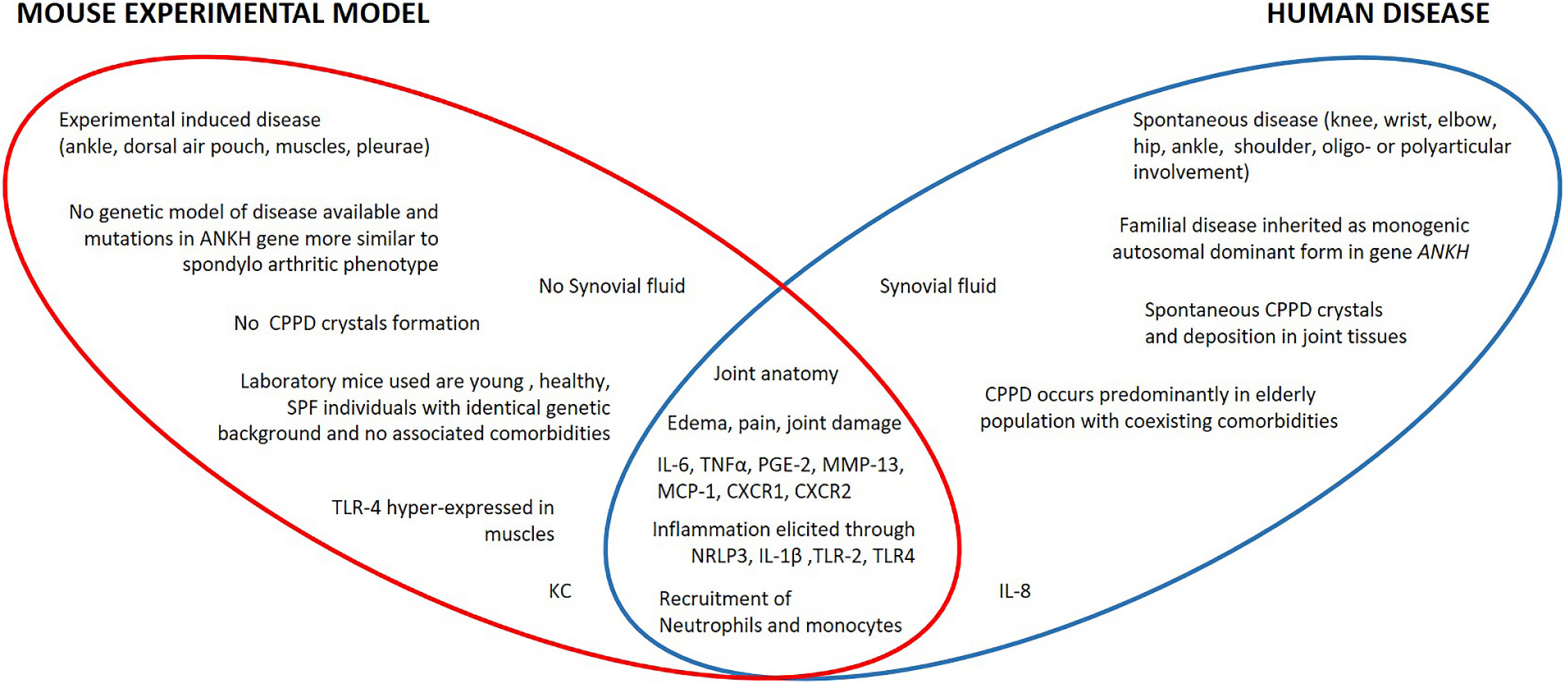
Specific and shared characteristics of human CPPD and corresponding mouse models. In mice, no spontaneous CPPD and CPP crystals formation has ever been observed. In humans, inflammation occurs at the joint level; in mice, induced inflammation is carried out in the ankle or in extra-articular anatomical districts such as the dorsal air pouch or in the intraperitoneal visceral compartment that has no homologies with the human pathology. Furthermore, laboratory rodent strains differ significantly for clinical conditions from the human population affected by CPPD. Apart from some species-specific molecular mediators such as IL-8 or KC and differences in the basal composition of leukocytes, humans and mice share the same molecular and histopathological mechanisms of CPP-induced inflammation.

CPP crystal-induced inflammation models reproduce some important aspects of the disorder and provide key information on the molecular pathophysiology of human disease (Figure 1). They are relatively simple, fast to realize, and useful for testing new therapeutical approaches. However, they only reproduce what the crystals cause but not their formation, thus not providing useful information on the mechanisms of the early stage of the disease. Furthermore, no experimental studies have been conducted to characterize the specific differences in the various clinical manifestations associated with CPP deposition. Finally, none of the models used to date consider the frequent presence of osteoarthritis or associated conditions such as hyperparathyroidism, hemochromatosis, or hypomagnesaemia observed in CPPD patients. Therefore, one of future efforts should be to develop animal models that reproduce the impact of these concomitant diseases to investigate their role in CPPD.

## Author contributions

RL: Conceptualization, Writing – original draft. AS: Conceptualization, Writing – review & editing.
